# The Mechanism of *Ranunculus ternatus* Thunb. against Esophageal Squamous Cell Carcinoma Based on Network Pharmacology and Experimental Verification

**DOI:** 10.2174/0113862073376779250410054456

**Published:** 2026-04-22

**Authors:** Michen Deng, Yanhong Deng, Zugui Tang, Wenqiang Nie, Yi Lu, Xicheng Wang, Zhe Wang, Yi Kong

**Affiliations:** 1 Department of Oncology, JingMen People's Hospital, Jingmen 448000 Hubei, China;; 2 Department of Pharmacy, The Third Affiliated Hospital of Guangzhou Medical University, Guangzhou 510150, China;; 3 Department of Oncology, The First Affiliated Hospital of Guangdong Pharmaceutical University, Guangzhou 510080, China;; 4 Department of Pharmaceutical Sciences, Shenzhen University of Advanced Technology (SUAT), Shenzhen 518107, China;; 5 Department of Traditional Chinese Medicine, The First Affiliated Hospital of Guangdong Pharmaceutical University, Guangzhou 510080, China;; 6 Guangdong Pharmaceutical University, Guangzhou 510006, China

**Keywords:** Esophageal squamous cell carcinoma, Ranunculus ternatus Thunb., network pharmacology, PI3K/Akt signaling pathways, esophageal cancer

## Abstract

**Introduction:**

Esophageal Squamous Cell Carcinoma (ESCC) remains a significant global health challenge, underscoring the urgent need for the development of innovative therapeutic approaches. Ranunculus ternatus Thunb., a traditional herb, exhibits potential anticancer properties, but its mechanisms against ESCC remain poorly understood. This study integrates network pharmacology and experimental validation to explore the therapeutic effects of ethyl acetate extract of *Ranunculus ternatus* Thunb. (RTE).

**Methods:**

Potential targets of RTE and ESCC were screened using public databases. A Protein-Protein Interaction (PPI) network was constructed to identify key targets, followed by GO and KEGG pathway enrichment analyses. The predicted mechanisms were validated using in vitro assays, including cell proliferation analysis and western blot assay in ESCC cell lines.

**Results:**

Network pharmacology analysis identified 274 potential targets, with 14 key genes implicated in the therapeutic effects of RTE. GO analysis revealed significant involvement in the inflammatory response and apoptotic signaling pathways. KEGG pathway analysis highlighted the MAPK, Relaxin, and PI3K/Akt signaling pathways as critical mechanisms. *In vitro* experiments demonstrated that RTE significantly inhibited the proliferation of EC-109 and TE-13 cells by modulating the MAPK/ERK and PI3K/Akt pathways.

**Discussion:**

The study reveals that active compounds of RTE target MAPK/ERK and PI3K/Akt pathways, aligning with prior evidence. However, future studies should explore animal models to confirm efficacy.

**Conclusion:**

This study provides a comprehensive understanding of the molecular mechanisms underlying the anticancer effects of RTE against ESCC. These findings underscore the potential of RTE as a promising natural compound for ESCC treatment.

## 1 INTRODUCTION

Esophageal Cancer (EC) is the 11^th^ most common cancer and the seventh leading cause of cancer death globally, with an estimated 511,000 new cases and 445,000 deaths in 2022, representing a significant global health challenge [1]. Among these, Esophageal Squamous Cell Carcinoma (ESCC) accounts for 90% of EC worldwide, particularly in high-risk regions such as Eastern Asia and Eastern Africa [2]. Early-stage tumors should undergo surgical resection, while locally advanced tumors should be treated with a systemic treatment regimen, including chemoradiotherapy, targeted therapy, immunotherapy, and palliative care. Standard chemotherapy, typically cisplatin with 5-FU or paclitaxel, remains unchanged due to the limited success of newer drugs in addressing side effects and resistance. Although molecular targeted therapies and immune checkpoint inhibitors show promise in early-stage EC, resistance often reduces their long-term effectiveness [3]. Despite treatment advances, ESCC continues to exhibit a high fatality rate, with overall 5-year survival rates ranging from 15% to 25% [4]. These limitations highlight the necessity for the advancement of novel therapeutic strategies that offer improved efficacy and reduced side effects.

Traditional Chinese Medicine (TCM) remedies, a form of complementary and alternative medicine, are frequently utilized in conjunction with antineoplastic drugs for the treatment of diverse cancer types. These remedies are increasingly acknowledged as potential therapeutic agents due to their favorable characteristics, including minimal side effects, low resistance development, and prolonged efficacy. Recent studies have categorized the effects of natural products on EC into several anti-cancer mechanisms, including the induction of apoptosis, inhibition of angiogenesis, and suppression of metastasis [5-16]. These findings underscore the potential of natural products to act as candidates for treating EC, presenting a promising direction for future research and development in this field. *Ranunculus ternatus* Thunb. (Ranunculaceae) is a plant species belonging to the Ranunculus genus, which has been included in the Chinese pharmacopoeia since 1990. It is predominantly found in the Henan and Anhui regions of China and has been utilized in traditional Chinese medicine for the management of tuberculosis, faucitis, and neck scrofula [17, 18]. The ethyl acetate extract, saponins, and polysaccharides are derived from *Ranunculus ternatus* Thunb. demonstrated anti-tumor effects both *in* *vitro* and *in vivo* in various cancers [19-27]. *Ranunculus ternatus* Thunb. is widely used clinically and has been approved for the treatment of cancer [28, 29]. Currently, it has been further developed for medicinal use in Japan for the treatment of breast cancer, demonstrating significant efficacy [30]. Nevertheless, the specific active ingredients and molecular targets of this compound have yet to be fully elucidated.

Network pharmacology, which clarifies the synergistic effects and underlying mechanisms of multi-component and multi-target agents using the analysis of networks, is a suitable approach to reveal the functional mechanisms of multi-target drugs [31, 32]. In this study, we first screened the active ingredient *in vitro*, then through network pharmacology to uncover the pharmacological mechanism of the compounds of *Ranunculus ternatus* Thunb. on ESCC and further verified through biological experiments.

## 2 MATERIALS AND METHODS

### 2.1 Network Pharmacology-Based Analysis

#### 2.1.1 Screening of Bioactive Components of RTE and Collection Targets

The active components of the RTE extract were identified through a review of the literature. The chemical composition information, such as molecular structures, canonical smiles, and 'SDF' files, was obtained from product databases, including PubChem (https://pubchem.ncbi.nlm.nih.gov/) and ZINC (http://zinc15.docking.org). Subsequently, the targets of the bioactive components were predicted using public databases such as TCMSP (http://tcmspw.com), PubChem (https://pubchem.ncbi.nlm.nih.gov/), PharmMapper (http://www.lilab-ecust.cn/pharmmapper/), TargetNet (http://targetnet.scbdd.com/), and Similarity Ensemble Approach database (SEA,http://sea.bkslab.org/). Additionally, the targets were standardized to unified gene names using the protein database UniProt (http://www.uniprot.org/uploadlists/). The target protein names were entered into UniProtKB, with the organism limited to “Homo sapiens,” prior to the retrieval of the official symbol.

#### 2.1.2 Acquisition of Intersection Genes and Network Construction

The search for "esophageal squamous cell carcinoma" related genes was conducted using the GeneCard database (https://www.genecards.org/). The intersection of predicted RTE targets and ESCC-related genes was obtained through Venny 2.1.0. Subsequently, a network titled "Components-targets-ESCC" was constructed using Cytoscape software. Protein-Protein Interaction (PPI) analysis of the intersection genes was carried out using the String software, with a focus on data with a confidence level exceeding 0.9 The network was visualized and analyzed using Cytoscape software in this research study.

#### 2.1.3 GO and KEGG Pathway Analysis

The key genes underwent GO and KEGG pathway enrichment analyses through the utilization of R software. Following the conversion of input molecular list IDs, the enrichment analysis was conducted using the clusterProfiler package (species: Homo sapiens, ID transformation library (package): org.Hs.eg.db), and the resulting enrichment analysis outcomes were visually represented using the ggplot2 package [33, 34].

#### 2.1.4 Preparation of R. ternatus extracts

The dried root powder of R. ternatus (0.2 kg) was extracted 3 times with 95% ethanol at 78℃. Following filtration and evaporation, the residue was suspended in H_2_O and subsequently fractionated with petroleum ether (60-90°C), ethyl acetate, and n-BuOH three times. This process yielded the R. ternatus petrloeum ether extract (RTP, 7.7 g), the R. ternatus ethyl acetate extract (RTE, 3.3 g), and the R. ternatus n-BuOH extract (RTB, 10.9 g). The three R. ternatus extracts were then dissolved in dimethyl sulfoxide at a concentration of 200 mg/mL.

#### 2.1.5 Cell Culture

The Human esophageal squamous cell carcinoma cell lines EC-109, TE-13, and TE-1 were purchased from the Cell Bank of Type Culture Collection of the Chinese Academy of Sciences (Shanghai, China). All cell lines were cultured in RPMI-1640 (Gibco; Thermo Fisher Scientific, Inc., Waltham, MA, USA) supplemented with 10% fetal bovine serum (FBS; Gibco; Thermo Fisher Scientific, Inc.), 100 U/ml of penicillin, and 100 µg/ml of streptomycin at 37°C in a humidified atmosphere containing 5% CO_2_.

#### 2.1.6 Cell Viability Assay

The cell viability was evaluated using the 3-(4,5-dime-thylthiazol-2-yl)-2,5-diphenyltetrazolium bromide (MTT) assay, which relies on the metabolic activity of viable cells to convert MTT into a formazan product. Specifically, cells were plated at a density of 5000 cells per well in 96-well plates and allowed to adhere overnight. Following adhesion, the cells were exposed to various concentrations (0, 50, 100, 200, 400, 600, and 800μg/mL) of RTE, RTP, and RTB (0, 200, 400, 600, 800, 1000, and 2000μg/mL) for 48 hours. Subsequently, MTT was added to each well, and the cells were incubated for an additional 4 hours. The colored formazan product was determined photometrically at 570 nm in a multi-well plate reader (Bio-Rad Laboratories, Hercules, CA).

#### 2.1.7 Western Blotting

EC-109 and TE-13 cells were seeded at a density of 5 × 10^5^ cells per dish in 60-mm culture dishes and incubated for 24 h. Following treatment with the IC_50_ concentration of RTE for an additional 48 hours, the cells were lysed in RIPA lysis buffer supplemented with a protease-inhibitor cocktail on ice for 15 minutes. The lysed cell suspensions were then centrifuged at 12,000 ×g at 4°C for 15 minutes, and the resulting supernatants containing soluble cellular proteins were collected and stored at -80°C until further analysis. BCA protein assay kits were utilized for the quantification of protein concentration. Subsequently, 20 μg of protein was reconstituted in sample buffer and subjected to separation *via* 10% SDS-polyacrylamide gel electrophoresis. The resolved proteins were then transferred onto a PVDF membrane, which was subsequently blocked using 5% milk in tris-buffered saline/0.1% Tween20. Following this, the membranes were probed overnight at 4 °C and immunodetection was carried out using primary antibodies, including anti-rabbit Phospho-p44/42 MAPK antibody (1:1000), anti-rabbit Phospho-Akt antibody (1:1000), anti-rabbit c-Myc antibody (1:1000), anti-rabbit HSP90 antibody (1:1000), and anti-rabbit β-actin antibody (1:1000). The membranes were incubated with a secondary antibody conjugated to peroxidase (1:2000, all from Cell Signaling Technology, Beverly, MA) and subsequently analyzed using the ECL Western Blotting Detection System (GE Healthcare, Little Chalfont, UK) to visualize signals, which were captured on X-ray film (Fuji Photo Film, Tokyo, Japan).

#### 2.1.8 Statistical Analysis

The experiments were conducted with a minimum of three repetitions each. Prism statistical software (Graph Pad Software, San Diego, CA) was utilized to calculate the half Inhibitory Concentration (IC_50_) values of the drugs. The figures present data as mean ± S.E.M., while the tables display data as mean ± SD. The statistical analysis was performed using SPSS 25.0 software (SPSS Inc., Chicago, IL), with Student's two-tailed t-test used for comparing data between two groups and Repeated measures ANOVA or One-way ANOVA for comparing data between three or more groups. *P* < 0.05 were considered statistically significant.

## 3 RESULTS

### 3.1 Identification of Potential Bioactive Compounds and Targets of RTE

A total of 29 active components from the RTE were searched in the published literature [28, 35-42] (Table **S1**). After eliminating the overlapping proteins, 461 associated proteins were obtained for subsequent analysis.

### 3.2 Construction of the Volatile Component-Target-Disease Pathway

A total of 4838 related targets were obtained from GeneCards (https://www.genecards.org/) and OMIM database using the search terms “esophageal squamous cell carcinoma.” Following the removal of duplicate proteins, a set of 274 shared targets between RTE and esophageal squamous cell carcinoma was identified. The regulatory network of RTE acting on ESCC was constructed using Cytoscape software version 3.7.2 The compound-compound target-disease network, illustrated in Fig. (**1**), consisted of 305 nodes (comprising 29 component nodes, 274 common targets, RTE, and ESCC) and 1640 edges. The network analysis indicates that a single component may interact with multiple targets, while multiple targets may be affected by a single component. Degree centrality is the measure of how important a node is at linking poorly connected parts of the network [43]. Among the 84 nodes identified with an average degree value of ≥8.82, comprising 29 compounds and 55 genes (Table **S2**), hexadecanoic acid, amentoflavone, and phillygenin are likely key components of RTE responsible for its anti-tumor properties.

### 3.3 PPI Network Analysis

Furthermore, to elucidate the systemic and pharmacological mechanism of RTE in the treatment of ESCC, we initially constructed and visualized a Protein-Protein Interaction (PPI) network comprising 274 putative targets. The network consists of 247 targets and 1279 edges, and an average value of a degree is 10.36 (Fig. **2**). In addition, 77 core targets were identified in descending order (Table **S3**). These hub targets included AKT1、HSP90AA1、EGFR、MAPK1, and TNF， indicating that they serve as vital targets of RTE in treating ESCC.

### 3.4 Key Genes

Finally, the above two network diagrams were combined to obtain 14 key genes of RTE acting on ESCC (Fig. **3**).

### 3.5 GO Functional Enrichment Analysis and KEGG Pathway Enrichment Analysis

Furthermore, to identify the biological characteristics of putative targets of RTE on ESCC in detail, the GO and pathway enrichment analyses of involved targets were conducted *via* R software (4.2.1 version). The results of GO enrichment analysis showed that the therapeutic effect of RTE on ESCC was mainly related to biological processes such as regulation of inflammatory response, response to lipopolysaccharide, and regulation of apoptotic signaling pathway. The cellular components include the vesicle lumen, secretory granule lumen, and cytoplasmic vesicle lumen. The molecular functions included RNA polymerase II-specific DNA-binding transcription factor binding, transcription coregulator binding, and ligand-activated transcription factor activity (Fig. **4**). As demonstrated in Fig. (**5**), the results of the KEGG pathway analysis suggested that RTE may exert anti-tumor effects through MAPK signaling pathway, Relaxin signaling pathway, and PI3K/Akt pathway, *etc*.

### 3.6 The Cytotoxicity of RTP and RTE on ESCC Cells

Moreover, to evaluate the cytotoxic potential of three different extracts derived from *R*. ternatus, IC_50_ values were determined. The inhibitory effect of RTB on the viability of three ESCC cell lines was observed to be less than 50% at a concentration of 2000 µg/mL. The MTT assay analysis revealed that treatments with RTE and RTP suppressed ESCC cell viability, with no statistically significant differences in IC_50_ values between the two extracts within each cell line, as detailed in Table **1**. Notably, the IC50 values for both RTE and RTP were significantly lower in EC-109 cells compared to the other two cell lines, suggesting an increased cytotoxic efficacy in this particular cell line (*P*<0.05).

### 3.7 RTE Suppresses MAPK/ERK Pathway and PI3K/Akt Pathway

Based on the results of network pharmacology, it is imperative to elucidate the pathways associated with these three proteins-. The results indicated a significantly decreased expression of p-ERK1/2, p-Akt, HSP90, and c-Myc in both EC-109 and TE-13 cell lines following treatment with RTE compared to the control group. Furthermore, no significant differences were observed in the expression levels of total ERK1/2 and Akt between groups (Fig. **6**).

## 4 DISCUSSION

ESCC is a prevalent malignancy associated with various proteins and signaling pathways that contribute to its development and progression [44-47]. TCM, which consists of a wide variety of compounds, exhibits extensive pharmacological effects that target multiple pathways and molecular targets, thereby offering potential therapeutic benefits for ESCC treatment [48]. However, the complexity of TCM poses challenges for elucidating its underlying mechanisms. The application of network pharmacology, which combines systems biology and network analysis methodologies, may provide a promising strategy for exploring the complex mechanisms of action of TCM in the treatment of ESCC. The extracts of *Ranunculus ternatus* Tunb. have shown their anti-tumor activity in many studies [20, 23, 24], but the anti-tumor mechanism of it has not yet been elaborated. The primary objective of this study was to investigate the impact of RTE on ESCC and validate the mechanisms proposed by network pharmacology through *in vitro* experimentation.

The findings from network pharmacology indicate that hexadecanoic acid, amentoflavone, and phillygenin are principal active constituents of RTE in the treatment of ESCC. Hexadecanoic acid also referred to as palmitic acid, is characterized as a saturated long-chain fatty acid with a straight-chain structure consisting of sixteen carbon atoms [49]. Research has shown that palmitic acid can induce cell cycle arrest and promote apoptosis in human neuroblastoma cells and breast cancer cells. Additionally, it has been found to inhibit the proliferation of hepatoma cells by altering membrane fluidity and interfering with glucose metabolism [50-52]. In prostate cancer cells, palmitic acid has been shown to suppress proliferation and metastasis by inhibiting the PI3K/Akt pathway [53]. Furthermore, palmitic acid has been demonstrated to inhibit the growth and metastasis of gastric cancer by blocking the STAT3 signaling pathway [54]. Amentoflavone, a biflavonoid compound, exhibits anti-tumor activity in various types of cancer. Studies have demonstrated its involvement in numerous biological processes, such as cell proliferation, apoptosis, cell cycle regulation, migration, and invasion [55-58]. Phillygenin has been reported to demonstrate anti-tumor activity in drug-resistant human esophageal cancer, pancreatic cancer, osteosarcoma, and non-small cell lung cancer [59-62].

The findings from the PPI network analysis revealed 77 core targets associated with RTE in the treatment of ESCC, such as AKT1, HSP90AA1, EGFR, MAPK1, and TNF. These targets are intricately linked to cellular processes, including proliferation, cell cycle regulation, and apoptosis, which are crucial for the therapeutic efficacy of RTE in ESCC [63-65]. The GO enrichment analysis of these hub targets suggests that RTE primarily engages in biological processes such as regulation of inflammatory response, response to lipopolysaccharide, and regulation of apoptotic signaling pathway. Analysis of the KEGG pathway results showed that RTE is mainly involved in the MAPK signaling pathway, Relaxin signaling pathway, PI3K/Akt pathway, *etc*. These pathways are closely related to the growth and metabolism of tumors, indicating that RTE may regulate the above pathways through hub targets, particularly MAPK1 and HSP90, thereby inhibiting tumor progression.

In order to elucidate the molecular mechanism of RTE in the treatment of ESCC, we conducted *in vitro* cell experiments to validate the predicted outcomes. MTT assays were performed on EC109, TE-1, and TE-13 cells, revealing a dose-dependent anti-ESCC effect of both RTE and RTP. These findings suggest that RTE may hold promise as a potential therapeutic agent for ESCC. Previous research has indicated the anti-tumor properties of Ranunculus ternatus Thunb. Extracts, prompting our selection of RTE for further investigation in this study [20, 23, 24]. The network analysis revealed that the hub targets primarily participate in the MAPK signaling pathway. Considering the interconnectedness of signaling pathways and their downstream targets, as well as the relevance of closely associated targets identified in the literature on ESCC, ERK1/2, p-ERK1/2, AKT, p-AKT, HSP90, and c-Myc proteins were chosen for validation through Western blot experiments [66-72]. In comparison to the control group and the group treated with RTE in Eca-109 and TE-13 cells, a significant decrease in the expression of p-ERK1/2 and p-Akt was observed. Additionally, a slight reduction in HSP90 and c-Myc expression was noted in the RTE-treated group. These findings suggest that RTE has the potential to inhibit the MAPK/ERK and PI3K/Akt pathways, as well as downregulate the expression of oncogenic factors HSP90 and c-Myc, ultimately leading to the inhibition of cell proliferation in ESCC.

MAPK1 is one of the hub targets in our analysis， which is an important protein in the MAPK/ERK pathway. MAPK/ERK pathway is obviously the most well-studied signal transduction module, especially in the context of ESCC development [73]. Moreover, c-Myc is activated upon various mitogenic signals, such as *via* the MAPK/ERK pathway [74]. By modifying the expression of its target genes, c-Myc activation results in numerous biological effects, including cell proliferation, apoptosis, and differentiation. Under stimulation, ERK kinases migrate to the nucleus and phosphorylate their nuclear protein substrates. The anti-apoptosis response in many tumor cells is initiated through the ERK1/2-mediated pathway [75]. The results revealed that RTE significantly decreased the expression of p-ERK1/2. Therefore, RTE inhibited ESCC *via* the MAPK/ERK pathway.

Besides, Hsp90 is another hub target, which stabilizes various growth factor receptors [76] and some signaling molecules, including PI3K and Akt proteins. Thereby, inhibition of Hsp90 downregulates the PI3K/Akt pathway, resulting in apoptosis of cancerous and senescent cells [77]. PI3K/Akt pathway plays an important regulatory role in the process of tumor cell proliferation and anti-apoptosis [78]. Our results showed that RTE significantly down-regulated the expression of p-Akt and HSP90. Therefore, RTE weakens the proliferation of ESCC cells by inhibiting the PI3K/Akt pathway.

In terms of academic contributions, this study provides a novel insight into the molecular mechanisms of RTE against ESCC by integrating network pharmacology with experimental validation. Utilizing advanced computational tools to identify potential therapeutic targets, the research establishes a systematic framework for understanding how traditional herbal medicines, such as RTE, can be applied in cancer treatment. Moreover, the findings contribute to the growing body of research on the use of natural compounds in targeting critical signaling pathways involved in cancer progression. The validation of the MAPK/ERK and PI3K/Akt pathways as critical mediators in ESCC treatment further strengthens the potential of RTE as a promising therapeutic agent for cancer.

Despite the promising results, this study is subject to several limitations that warrant consideration. Firstly, although network pharmacology offers a comprehensive analysis of potential targets, the predictive model relies on existing public databases, which may not encompass all relevant targets or signaling pathways. Furthermore, while we have identified several key pathways and targets, the precise molecular mechanisms by which RTE influences these pathways remain unclear. Future research employing advanced techniques, such as proteomics or transcriptomics, could elucidate the molecular actions of RTE more thoroughly. Additionally, while our *in vitro* experiments utilizing EC-109 and TE-13 cell lines provide preliminary insights into the effects of RTE, it is essential to validate these findings *in vivo* using animal models of ESCC to confirm the therapeutic efficacy of RTE within a more complex biological framework. Furthermore, although the emphasis on the MAPK/ERK and PI3K/Akt pathways is of significant relevance, it is imperative for future research to investigate additional signaling pathways that may contribute to the anticancer effects of RTE. The Relaxin pathway, identified through KEGG analysis, emerges as a promising candidate for further exploration, given its involvement in various cancer types, despite its limited study in the context of ESCC.

## CONCLUSION

In this study, the results of network pharmacology and PPI network showed that MAPK1 and HSP90 were the key targets of RTE against ESCC. Through network pharmacology and experiments *in vitro*, RTE was verified against ESCC *via* the MAPK/ERK pathway and PI3K/Akt pathway, which may offer a useful tool to clear the molecular mechanism of RTE on ESCC treatment. However, further research is essential to enhance our understanding of its underlying mechanisms and evaluate its clinical applicability. Future investigations should focus on elucidating the precise molecular interactions between RTE and its targets, validating these findings *in vivo*, and exploring the broader therapeutic potential of RTE across various cancer types.

## AUTHORS' CONTRIBUTIONS

The authors confirm their contributions to the paper as follows: study conception and design: Y.K.; data collection: Y.D., Y.L.; analysis and interpretation of results: W.N.; manuscript drafting: Z.W., X.W.; validation: Z.T.; methodology: M.D. All authors reviewed the results and approved the final version of the manuscript.

## Figures and Tables

**Fig. (1):**
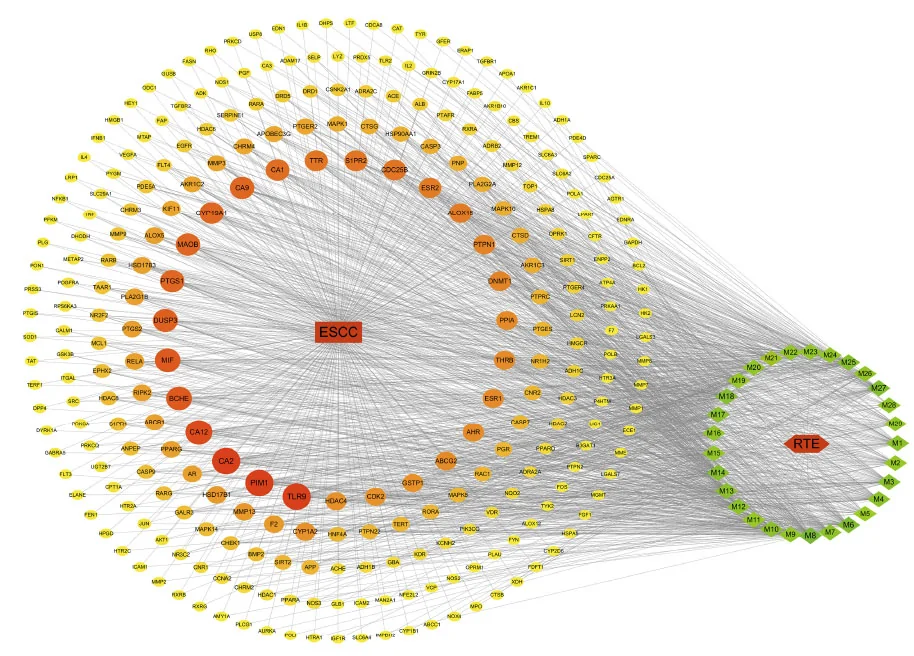
Component-target network. Ellipses represent active ingredients in RTE. Diamonds represent targets of RTE and ESCC. Edges represent the interaction between ingredients and targets.

**Fig. (2):**
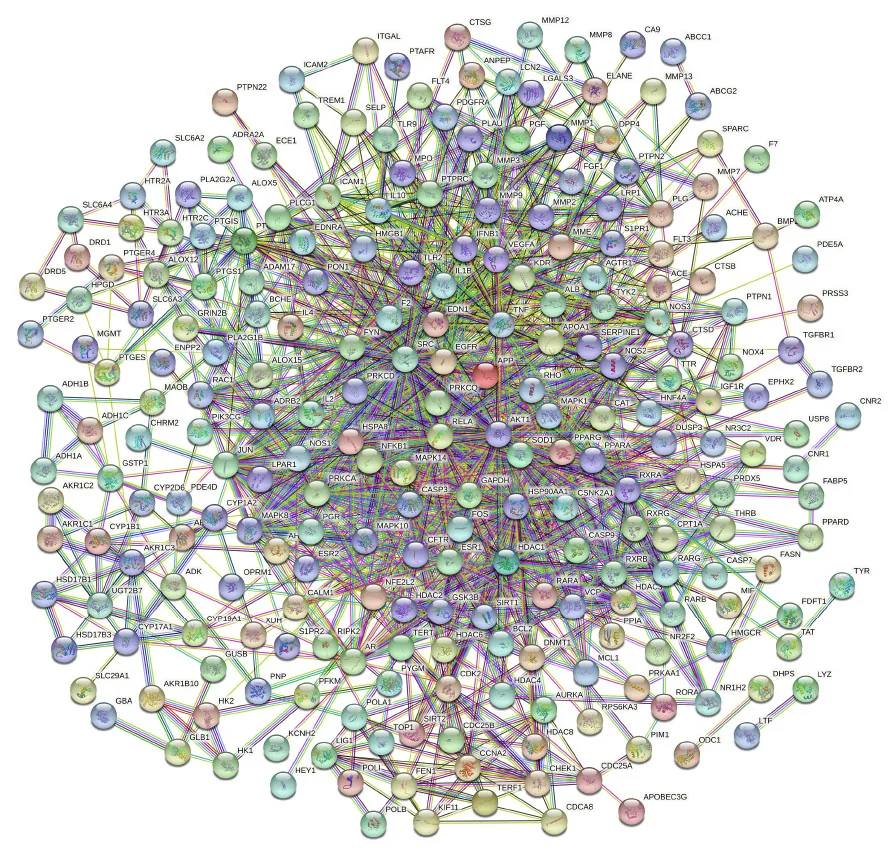
Protein-protein interaction network.

**Fig. (3):**
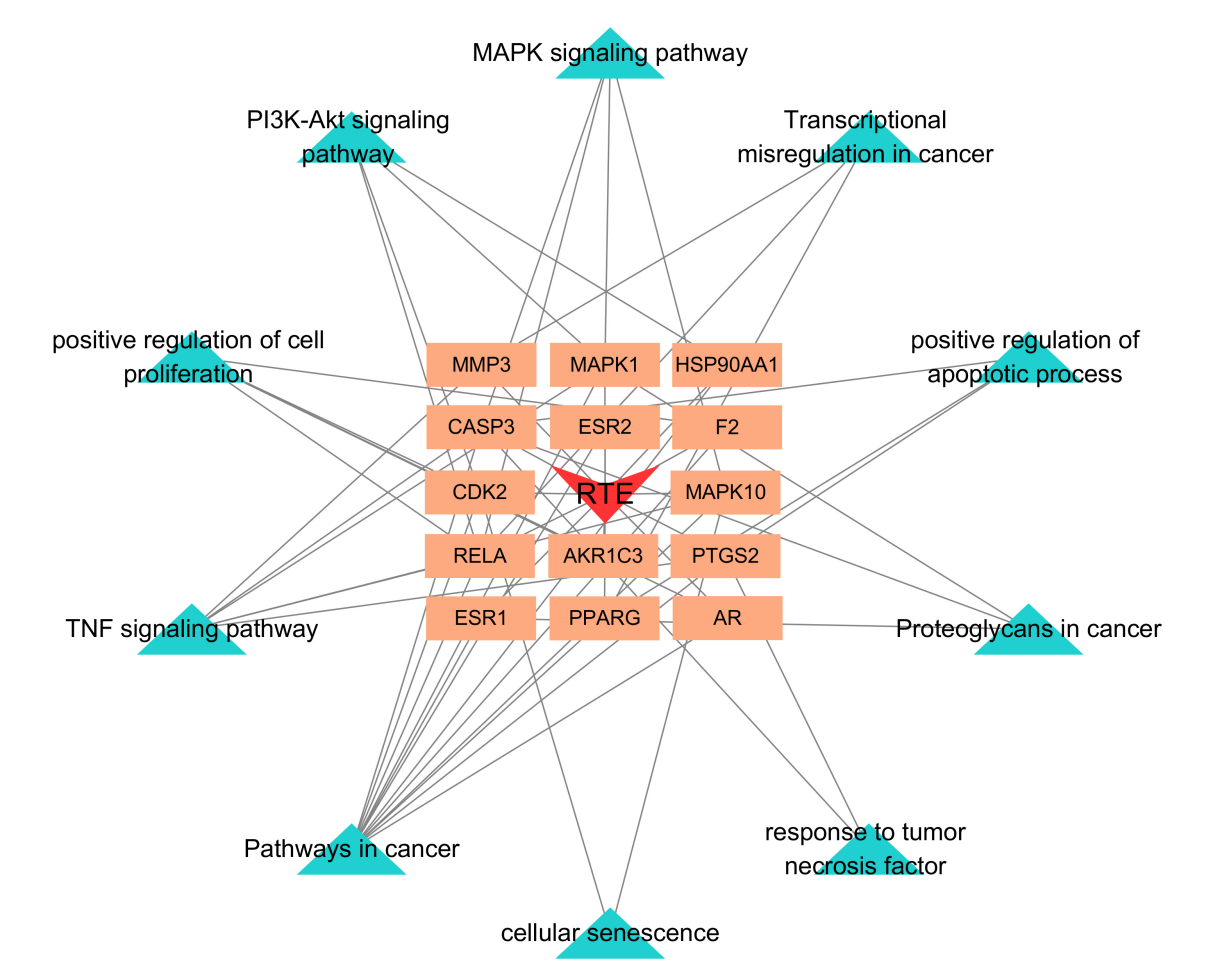
Fourteen intersecting potential bioactive compounds among the top compounds in PPI and MOL-Gene.

**Fig. (4):**
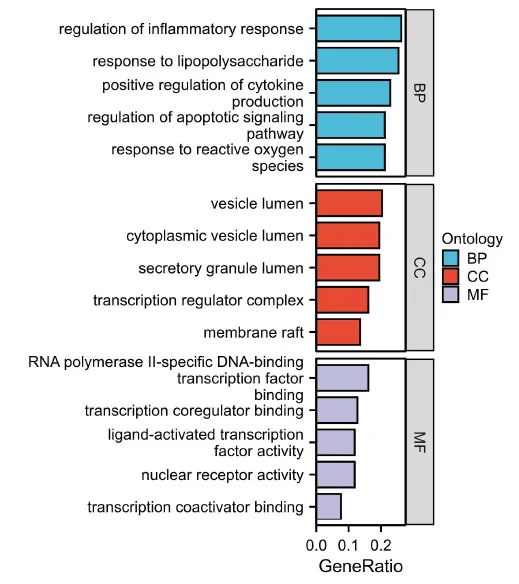
GO Enrichment Analysis.

**Fig. (5):**
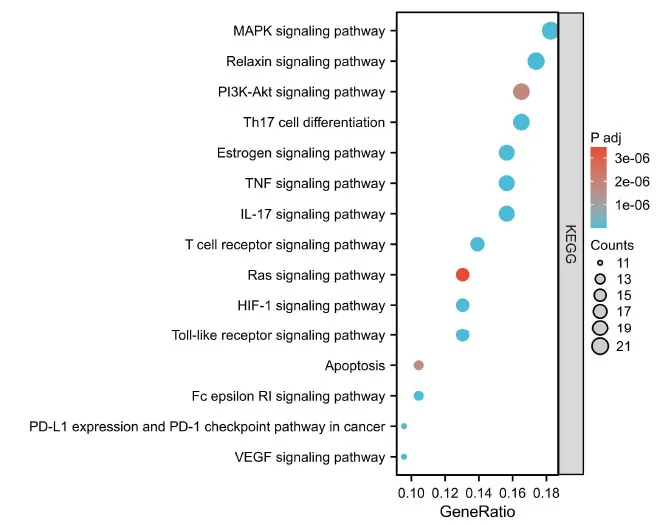
KEGG pathway enrichment analysis of targets. The Y-axis represents significant pathways of the target genes, and the X-axis shows the rich factor.

**Fig. (6):**
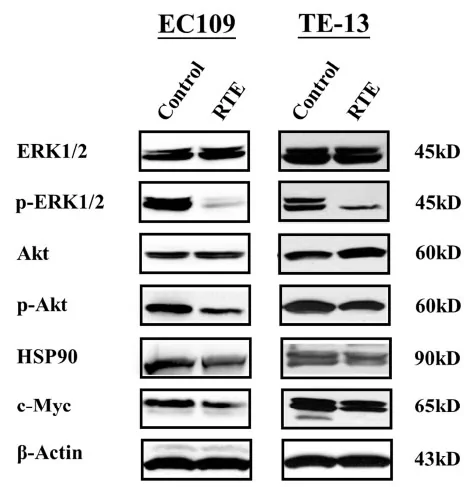
The expression of ERK1/2, p-ERK1/2, Akt, p-Akt, HSP90, and c-Myc proteins was determined by Western blot analysis.

**Table 1:** RTE and RTP suppressed the viability of ESCC cells.

** **	**IC_50_ (μg/mL)**		
** **	**TE-1**	**TE-13**	**EC-109**
RTE	220.8 ± 61.9	361.3 ± 9.5	90.2 ± 14.0^***^
RTP	298.9 ± 32.2	270.2 ± 51.4	100.2 ± 3.4^*^

**Note:** Each cell was treated for 48 h with three extracts of RT (0–2000 mg/mL). Cell viability was then determined by 3-(4,5-dimethylthi-azol-2-yl)-2,5-diphenyltetrazolium assay. IC_50_ values were calculated with Prism software. Data are presented as Mean ± SEM. (n=3) of a representative experiment performed in triplicate. *** presented *a P*-value less than 0.001, * presented a *P*-value less than 0.05.

## Data Availability

The original contributions presented in the study are included in the article and supplementary material. Further inquiries can be directed to the corresponding authors.

## LIST OF ABBREVIATIONS

ESCC: Esophageal Squamous Cell Carcinoma
GO: Gene Ontology
KEGG: Kyoto Encyclopedia of Genes and Genomes
RTE: Ranunculus ternatus Thunb.
