# Supplementary material for: The Mechanism of Ranunculus ternatus Thunb. against Esophageal Squamous Cell Carcinoma Based on Network Pharmacology and Experimental Verification
Source: Comb Chem High Throughput Screen. 2026 Apr 22;29(11):1598–609. doi: 10.2174/0113862073376779250410054456 (PMC13673438; doi:10.2174/0113862073376779250410054456)
Supplement: Supplementary file 1 [file CCHTS-29-11-1598_SD1.pdf]

Supplementary Material

The Mechanism of *Ranunculus ternatus* Thunb. against Esophageal Squamous Cell Carcinoma Based on Network Pharmacology and Experimental Verification

Michen Deng[1], Yanhong Deng[2], Zugui Tang[3,4], Wenqiang Nie[5], Yi Lu[6], Xicheng Wang[3], Zhe Wang[3*] and Yi Kong[4,*]

[1]*Department of Oncology, JingMen People's Hospital, Jingmen 448000 Hubei, China;* [2]*Department of Pharmacy, The Third Affiliated Hospital of Guangzhou Medical University, Guangzhou 510150, China;* [3]*Department of Oncology, The First Affiliated Hospital of Guangdong Pharmaceutical University, Guangzhou 510080, China;* [4]*Faculty of Pharmaceutical Sciences, Shenzhen University of Advanced Technology (SUAT), Shenzhen 518107, China;* [5]*Department of Traditional Chinese Medicine, The First Affiliated Hospital of Guangdong Pharmaceutical University, Guangzhou 510080, China;* [6]*Guangdong Pharmaceutical University, Guangzhou 510006, China*

Table S1. Identification of Potential Bioactive Compounds of RTE.

Number	Name	Pubchem ID	CAS ID	Molecular Formula
M1	3，4-Dihydroxybenzaldehyde	8768	139-85-5	C7H6O3
M2	5-Hydroxymethylfurfural	237332	67-47-0	C6H6O3
M3	Amentoflavone	5281600	1617-53-4	C30H18O10
M4	Bilobetin	5315459	521-32-4	C31H20O10
M5	Daucosterol	5742590	474-58-8	C35H60O6
M6	Dibutyl phthalate	3026	84-74-2	C16H22O4
M7	Henicosanoic acid methyle ester	22434	6064-90-0	C22H44O2
M8	Hexadecanoic acid	985	57-10-3	C16H32O2
M9	Isoginkgetin	5318569	548-19-6	C32H22O10
M10	Kayaflavone	9894522	481-45-8	C33H24O10
M11	Linocaffein	14825516	17093-82-2	C15H18O9
M12	Methylparaben	7456	99-76-3	C8H8O3
M13	Montanic acid	10470	506-48-9	C28H56O2
M14	N-eicosanoic acid	10467	506-30-9	C20H40O2
M15	Phillygenin	3083590	487-39-8	C21H24O6
M16	Diethylhexylphthalate	7057919	117-81-7	C24H38O4
M17	Podocarpusflavone A	5320644	22136-74-9	C31H20O10
M18	Salicylic acid	338	69-72-7	C7H6O3
M19	Sternbin	1268276	51857-11-5	C16H14O6
M20	Syringic acid	10742	530-57-4	C9H10O5
M21	β-D-glucose	64689	492-61-5	C6H12O6
M22	3,4-Dihydroxyl-phenylalanine ethyl ester	170345	37178-37-3	C11H15NO4

M23	4-O-D-glucopyranosyl-p-coumaric acid	10604651	117405-48-8	C15H18O8
M24	5-hydroxymethylfurfuric acid	80642	6338-41-6	C6H6O4
M25	Glycerol β-steariate	79075	621-61-4	C21H42O4
M26	Robustaflavone-4′ -methyl ether	11757331	-	C31H20O10
M27	Ternatoside A	-	-	C15H26O9
M28	Ternatoside B	-	-	C19H28O10
M29	Glycerol β-palmitate	123409	23470-00-0	C19H38O4

Table S2. Key Compounds and Targets of RTE in "component-target" network.

Degree	Gene	Type	Degree	Gene	Type
78	Hexadecanoic acid	MOL	17	S1PR2	Gene
66	Dibutyl phthalate	MOL	17	TTR	Gene
64	Ternatoside A	MOL	16	ALOX15	Gene
62	Salicylic acid	MOL	16	ESR2	Gene
59	Glycerol β-steariate	MOL	15	DNMT1	Gene
58	N-eicosanoic acid	MOL	15	PTPN1	Gene
54	Amentoflavone	MOL	14	ABCG2	Gene
54	Montanic acid	MOL	14	AHR	Gene
54	Podocarpusflavone A	MOL	14	ESR1	Gene
52	3,4-Dihydroxybenzaldehyde	MOL	14	PPIA	Gene
52	Bilobetin	MOL	14	THRB	Gene
52	Kayaflavone	MOL	13	CDK2	Gene
50	Henicosanoic acid methyle ester	MOL	13	CYP1A2	Gene
49	Methylparaben	MOL	13	GSTP1	Gene
44	Diethylhexylphthalate	MOL	13	HDAC4	Gene
44	Isoginkgetin	MOL	12	F2	Gene
44	Syringic acid	MOL	12	HSD17B1	Gene
42	Robustaflavone-4′-methyl ether	MOL	12	MMP13	Gene
40	Glycerol β-palmitate	MOL	11	ABCB1	Gene
39	5-Hydroxymethylfurfuric acid	MOL	11	ALOX5	Gene
39	Phillygenin	MOL	11	AR	Gene
39	Sternbin	MOL	11	HSD17B3	Gene
38	5-Hydroxymethylfurfural	MOL	11	PLA2G1B	Gene
37	Linocaffein	MOL	11	PPARG	Gene
31	Daucosterol	MOL	11	PTGS2	Gene
29	β-D-glucose	MOL	11	RELA	Gene
27	3,4-Dihydroxyl-phenylalanine ethyl ester	MOL	11	RIPK2	Gene
25	CA2	Gene	10	AKR1C2	Gene

25	PIM1	Gene	10	APOBEC3G	Gene
25	TLR9	Gene	10	CHRM4	Gene
23	CA12	Gene	10	KIF11	Gene
21	4-O-D-glucopyranosyl-p-coumaric acid	MOL	10	MMP3	Gene
21	BCHE	Gene	10	PTGER2	Gene
21	MIF	Gene	9	AKR1C3	Gene
20	DUSP3	Gene	9	CASP3	Gene
19	CA9	Gene	9	CTSD	Gene
19	CYP19A1	Gene	9	CTSG	Gene
19	MAOB	Gene	9	HSP90AA1	Gene
19	PTGS1	Gene	9	MAPK1	Gene
19	Ternatoside B	MOL	9	MAPK10	Gene
18	CA1	Gene	9	PLA2G2A	Gene
17	CDC25B	Gene	9	PNP	Gene

Table S3. Key genes of PPI Network Analysis.

Degree	Gene	Degree	Gene	Degree	Gene
62	AKT1	25	PTGS2	15	PRKCD
55	HSP90AA1	24	IL10	15	RARA
53	EGFR	23	AR	15	IFNB1
50	SRC	22	PLCG1	14	CCNA2
47	MAPK1	21	IL2	14	ESR2
46	JUN	21	FOS	14	MPO
44	TNF	20	TLR2	14	APOA1
38	VEGFA	20	ICAM1	14	HDAC2
37	RELA	20	PTPRC	14	CHEK1
35	ALB	20	PPARA	13	SOD1
35	ESR1	19	HSPA8	13	TERT
35	IL1B	19	NOS2	13	HSPA5
34	GAPDH	19	FYN	13	MMP1
33	MAPK8	19	IL4	13	HMGB1
32	MAPK14	19	PLG	12	AKR1C3
31	CASP3	19	HDAC3	12	RARB
30	APP	18	CAT	12	RARG
30	MMP9	18	RAC1	12	IGF1R
28	PPARG	18	RXRB	12	FEN1
28	RXRA	18	CDK2	12	PGR
27	NOS3	18	KDR	11	ACE
27	SIRT1	17	F2	11	NFE2L2

27	HDAC1	17	MMP2	11	BCL2
26	EDN1	17	RXRG	11	TYK2
25	PRKCA	16	SERPINE1	11	MMP3
25	NFKB1	16	MAPK10		
